# Exploring the value of the learners’ perception of teaching effectiveness in informing faculty development needs: A mixed-methods study

**DOI:** 10.15694/mep.2020.000213.1

**Published:** 2020-09-30

**Authors:** Farah Otaki, Amar Hassan Khamis, Reem AlGurg, Mohamed Nasaif, Dave Davis, Nabil Zary

**Affiliations:** 1 Mohammed Bin Rashid University of Medicine and Health Sciences (MBRU); 2 Training and Development Centre

**Keywords:** Faculty Development, Student Engagement, Postgraduate, Undergraduate, Performance, Continuous Quality Improvement.

## Abstract

This article was migrated. The article was marked as recommended.

The evaluation of a faculty development intervention needs to start at the outset, and not as an afterthought. Moreover, it is important to evaluate the degree to which the predefined impact is attained as a result to the learning and development opportunity. This calls for the engagement of the ultimate receivers: the students, who are well-positioned to identify gaps in the teaching performance of their own instructors. Accordingly, this mixed-methods study aims at shedding light on a Continuous Quality Improvement system where feedback from undergraduate and postgraduate students is assembled to pinpoint faculty development needs, based on which professional development opportunities are devised.

Data was extracted from an annual survey that evaluates the students’ satisfaction. Qualitative data was thematically analysed, and quantitative data was analysed using SPSS.

The qualitative analysis resulted in six categories of recommendations, that were fit into two themes: dynamic between the instructors and students, and organization and delivery of the program. As for the quantitative analysis, the students indicated opportunities for improvement in the following two areas: student academic advising process (55.17%), and communication between instructors and students (50.59%).

The study concluded that there is added value in capturing and effectively assembling the learners’ perception of faculty performance. It raises the reliability of the implemented evaluation framework, and has the potential of improving the rigor of faculty development initiatives.

## Introduction

It makes sense to assume that instructors who spend time developing their teaching knowledge and skills will become more effective, and that this in turn will translate into better student outcomes. However, there are plenty of challenges in tracing the effects of the professional development of faculty members on student learning ( Steinert, 2017). That can explain why the research on this matter is patchy, relying mainly on self-reported measures. The evaluation of the faculty development intervention needs to start at the outset, and not as an afterthought, and it is crucial to capture multiple perspectives ( Clay *et al.*, 2013; Motola *et al.*, 2013; Cook-Sather and Felten, 2017).

The Kirkpatrick model, which is considered an industry standard in the learning and development world, highlights the importance of tackling four levels of evaluation ( Kirkpatrick and Kirkpatrick, 2006), namely: reaction, learning, behaviour, and results. The last level refers to the degree to which the predefined impact is attained as a result to the learning and development opportunity.

There are some studies that suggest that faculty development can have measurable impacts on teaching ( Berbano *et al.*, 2006; Burdick *et al.*, 2010; Wright, Mullen and Gardner, 2019). A few of these articles shed light on how self-reported learning gains, recorded right upon completion of a specific development opportunity, are congruent with the pre-set objectives of the opportunity itself ( Bewley and O’Neil, 2013; Ten Cate *et al.*, 2014). In other references, faculty members report looking back at past the development opportunities, describing positive changes over time around their teaching skills in alignment with the opportunities’ pre-set objectives ( Steinert, 2017).

This calls for the engagement of the ultimate receivers: the students, who are well-positioned to identify gaps in the teaching performance of their own instructors. Along the same lines, Bovill, Cook-Sather and Felten (2011) argued, using theoretical evidences, for including students as partners in pedagogical planning processes. They also presented examples where students have worked collaboratively in the learning design processes.

Despite the recent surge in attention towards involving students, one way or another, in pedagogical planning ( Beckman *et al.*, 2004; Wright, Mullen and Gardner, 2019), there are still no studies published in the literature that reflect upon processes and output of engaging students in identifying faculty development needs ( Cook-Sather and Felten, 2017; Wright, Mullen and Gardner, 2019). Accordingly, building upon the related theoretical foundation of O’Sullivan and Irby (2011), this study aims at shedding light on an experience where the feedback from undergraduate and postgraduate students was appraised and assembled as part of a university’s Continuous Quality Improvement (CQI) system to pinpoint the faculty development needs, based on which professional development opportunities can be devised. Accordingly, this study’s research questions are as follows:

• What opportunities for improvement, around faculty performance, do students perceive, and how do the perceptions of undergraduate and postgraduate students compare to each other?
• How do the perceived opportunities for improvement relate to one another?
• How can Higher Education Institutions utilize student feedback to identify faculty development needs and foster evidence-driven improvements?

## Methods

### Context of the study

This study was undertaken at the Mohammed Bin Rashid University of Medicine and Health Sciences (MBRU). MBRU is composed of two colleges: The Hamdan Bin Mohammed College of Dental Medicine (HBMCDM) and the College of Medicine (CoM). HBMCDM was the first college to be established at MBRU; this college offers several specialty dental postgraduate programs. The second college of the University is the CoM, which offers an undergraduate Bachelor of Medicine and Bachelor of Surgery (MBBS) program. The first batch of students of the MBBS program are expected to complete their studies in the Spring of 2021-2022. The targeted faculty development system is meant to become a cornerstone in the CQI set-up at MBRU.

### Study Design

A mixed-methods study design was adapted to systematically develop an understanding of the situation, and of student perceptions regarding faculty development needs. This study is characterized by a single phase, where existing qualitative and quantitative data was concurrently extracted and analysed. The triangulation of data, in terms of sources (i.e., students at different points in their educational trajectories) and types (i.e., qualitative and quantitative), is meant to raise the validity of the generated findings.

### Data Retrieval

The data was extracted from an annual survey that aims at assessing the satisfaction of students with their learning experience at the University. Participation in this annual data collection initiative is completely voluntary. In addition, the privacy and the data confidentiality of the students are protected, and no personal identifiers are recorded.

The dataset investigated was the one assembled throughout May 2018- covering for the Academic Year 2017-2018. In the respective academic year, the University was serving a total of 131 students. Out of those 131 students, 87 responded (i.e., response rate= 66.41%). Although 70% response rate would have been preferable, it is established in the literature that a 60% in a survey-based study is acceptable ( Baruch, 1999; Baruch and Holtom, 2008; Mundy, 2002; Morton *et al.*, 2012; Nulty, 2008). Among the 87 respondents, around 71% were from CoM, and the rest were from HBMCDM.

Each of the 87 participants were given a unique identification number. The unique identification numbers were complimented with ‘M’ for the 62 participants from CoM, and ‘DM’ for the 25 participants from HBMCDM (i.e., participants 1 through 62 are followed by ‘M’, and 63 through 87 by ‘DM’).

### Data Analysis

Qualitative Analysis

The extracted qualitative data was extracted from the students’ answers to the following five open-ended questions: Do you feel happy studying at this university? Please elaborate; Will you recommend studying at this university to your friend? Please elaborate; What can the university do to increase your level of happiness?; What do you like the most about studying at this university?; and What could be improved at this university?

All the qualitative data, related to the students’ learning environment and their perception of the faculty members’ educational role, was thematically analysed, where patterns across the datasets were systematically identified and reflected upon. The data collected from each of the two colleges was handled separately. For each of the two datasets, all the data that was not related, directly or indirectly, to opportunities for improvement in relation to the faculty teaching performance was excluded. The remaining data was segmented into meaningful statements. The text fragments that refer to the same aspect of the faculty performance were compiled together, labelling each with an all-encapsulating title. For example, the following two text segments: “...give the students more space to voice their opinion...” and “...maintain the student-centred approach as the university grows...”, along with other related fragments, were compiled within a repository entitled: “maximizing students’ engagement”. The resulting categorization schemes of the two colleges were mapped onto each other to compare the nature of the opportunities pinpointed by the two differing populations of students (i.e., dental medicine postgraduate students versus MBBS undergraduate students). The same themes (more or less) surfaced in the separate (yet concurrent) analyses.

Quantitative Analysis

As for the extracted quantitative data, it corresponded to six parameters: teaching methods, level of respect and support received from instructors, communication between instructors and students, opportunity to express opinion, student academic advising process, and overall educational experience at MBRU. Each of the abovementioned parameters were assessed by students against a Likert scale (1: Extremely Dissatisfied, 2: Dissatisfied, 3: Neutral, 4: Satisfied, and 5: Extremely Satisfied). The quantitative data was descriptively analysed using SPSS for Windows version 25.0. For each of the six quantitative parameters, the mean and standard deviation were calculated, along with the sum of the percentage of students selecting Neutral, Disagree, and Strongly Disagree (which the researchers took to imply perceived opportunities for improvement).

As for the inferential analyses in between colleges, they were conducted using the Mann-Whitney test. In addition, a general linear univariant analysis was conducted to assess the extent to which the perceived “Level of respect and support received from instructors” (which is a core institutional value) can be explained by changes in the perceived “Opportunity to express opinion”, “Communication between instructors and students”, “Teaching methods” “Student academic advising process”, and the “Overall educational experience at MBRU”.

## Results/Analysis

### Qualitative Data

The adapted multi-staged thematic analysis revealed that students in both colleges see opportunities for improvement around two themes:

### Dynamic between the instructors and students

The first theme refers to the dynamic between instructors and students, and includes: maximizing students’ engagement, improving the quality of feedback provided, and maintaining a positive attitude.

A lot of the extracted text fragments, such as the examples below, constituted a call to engage students more in their own learning process:

3M: “...give the students more space to voice their opinions...”

65DM: “...maintain the student-centred approach as the University grows...”

Other students highlighted the need for improving the quality of the provided feedback:

60M: “...hold regular feedback sessions where the student and professor sit together (one-to-one), and discuss the mistakes the student has done for the student to avoid repeating the same ones in the future, as a student and also as a physician...”

52M: “...provide more guidance, academic-wise, specifically with what is getting taught and what is important to know from that...”

85DM: “...provide more guidance for us to acquire effective techniques to know what is important- this would decrease stress levels around exam times...”

As for the last category of fragments within this theme, it refers to recommendations for the instructors to maintain a positive attitude:

72DM: “...understand the needs of the students...”

10M: “...encourage instructors to be friendlier with the students...”

41M: “...a few of the instructors can be more encouraging...”

### Organization and delivery of the program

The second theme was related to the organization and delivery of the program, and included: adhering to course guides, enhancing the coordination across the faculty members, and improving the teaching skills among faculty members.

It seemed that the students see the value of having a more thorough orientation at the beginning of every course, and of informing them ahead of time of all that the respective courses will entail and require, in order for students to effectively manage their own expectations.

56M: “...I would appreciate if things were a bit more organized. For example, last minute announcements and changes could sometimes be frustrating...”

The students also highlighted an opportunity for improvement around the coordination across faculty members:

21M: “...better coordination within courses, and across lectures within the same course...”

77DM: “...increase the level of coordination...”

27M: “...make sure all professors, teaching the same course, are on the same page...”

23M: “...clinicians need to be better oriented before assignment. Their outstanding expertise in the clinical realm does not necessarily mean that they can teach it well to others...”

Finally, the students also referred to enhancing the teaching skills among faculty members:

11M: “...improve the content of the presentations...”

64DM: “...put clear objectives at the beginning of each presentation...”

67DM: “...encourage instructors to be open to receiving feedback, and not to take matters personally...”

80DM: “...some of the seasoned instructors can mentor the ones at earlier stages in their academic careers...”

### Quantitative Data

Descriptive Analyses

As for the quantitative analysis of the data, generated by all the University students (undergraduates and graduates), it revealed that most of the students feel happy studying at MBRU and would recommend it to a friend (72%). Upon encouragement to think proactively and provide constructive feedback, the students perceived, as illustrated in Table 1, opportunities for improvement across the following areas: Student Academic Advising Process (55.17%), Communication between instructors and students (50.59%), and Opportunity to express opinion (45.97%). The percentage of the mean of each of the abovementioned variables falls somewhere between Neutral and Satisfied. As for the Teaching methods (41.39%), the Overall educational experience at MBRU (37.93%), and the Level of respect and support received from instructors (32.19%), they were perceived as satisfactory by the students with the following means: 3.60(0.93), 3.63(0.89), and 3.63(0.89)4(1.13), respectively.

**Table 1.** Descriptive Quantitative Analyses

Item of Satisfaction	Mean(SD)	Percentage of the Mean	Category
Teaching methods	3.60(0.93)	72	S
Level of respect and support received from instructors	4(1.13)	80	S
Communication between instructors and students	3.28(1.17)	66	N-S
Opportunity to express opinion	3.24(1.38)	65	N-S
Student academic advising process	3.18(1.24)	64	N-S
Overall educational experience at MBRU	3.63(0.89)	73	S
Total average:	20.85(5.05)

N= Neutral; S= Satisfied

Inferential Analyses

The undergraduate students, with a mean of satisfaction of 22(4.51), rated the overall educational experience at the University higher than the postgraduate students, with a mean of satisfaction of 18(5.3) (P= 0.001). Upon further analysing the data, it was revealed that the graduate students perceive the potentiality of improvement across all the six parameters under investigation, with emphasis on the following three: Opportunity to express opinion (76%), Teaching methods (69.69%), and Communication between the instructors and students (68%). As for the undergraduate students, they collectively identified only two areas of improvement: Student academic advising process (56.45%), and Communication between the instructors and students (43.34%). The results also show that 67.3% of the perceived “Level of respect and support received from instructors” can be explained by perceived “Opportunity to express opinion” and “Communication between instructors and students”, adjusted over the perceived “Teaching methods”, “Student academic advising process”, and “Overall educational experience at MBRU”.

## Discussion

This study uncovers that capturing the learners’ perception of faculty performance enables tackling all four levels of the Kirkpatrick model, which is expected to raise the reliability of the adapted evaluation framework. The added value of assessing learners’ satisfaction with the quality of education and systematically factoring their opinion into Performance Improvement (PI) initiatives is also referred to in this study. In parallel to the suggestions of O’Sullivan and Irby (2011), adapting such an evidence-driven CQI system has the potential of improving the rigor of faculty development initiatives.

The undergraduate and postgraduate students offer differing yet complimentary perspectives. The undergraduate students appear to be more emotional ( Ginns, Prosser and Barrie, 2007). Their expectations relate to their needs right now, and to their imagination of the real-world ( Ramsden, 2006). The post-graduate student ideas seem more practical, and their needs are based largely on what they expect to require as they progress in their careers ( Bennett *et al.*, 2014; Lindsay, Breen and Jenkins, 2010). These differences in perceptions could be attributed to the fact that post-graduate students are older, and have work experience (since it is a prerequisite to joining the respective post-graduate programs at MBRU), and hence are more mature, and their ideas are grounded in real-life experiences. These perspectives can be compared to that of artistic trends of two fundamental stages in the history of Western art, namely: realism and impressionism. The former is a mid to late 19 ^th^ century movement that claimed that the artists, such as Gustave Courbet and Edouard Manet, attempted to represent the world as it is, and similar to the postgraduate students, their perception is solidly focused on the subject matter ( Young, 2015; Zamora and Faris, 1995). As for the impressionists (e.g., Paul Cezanne and Claude Monet), their paintings revealed strong perceptual impressions of sunlight, colour, and shadow ( Little, 2004). They were not interested in telling stories and painting morals, but rather, similar to the undergraduate students, wanted to represent their sensory impressions ( Stewart, 2003).

Both perceptions offer plenty of insights ( Zamora and Faris, 1995; Samarakoon *et al.*, 2013; Brennan *et al.*, 2003). By appraising the perceptive value of the feedback of both groups of students, and deciding on a pragmatic, scientifically-sound manner to consolidate them, Higher Education Institutions can tailor PI initiatives, around faculty development or otherwise, which address both the short- and long-term needs of their students.

The results of the qualitative and quantitative data analyses validate and build upon each other. It seems that the most prominent opportunity for improvement, in terms of faculty development, is around communication (including but not limited to listening). Students want to be more engaged in their own learning process. All of which is in alignment with the literature around the subject matter ( Bovill, Cook-Sather and Felten, 2011; Ginns, Prosser and Barrie, 2007; Ramsden, 2006; Brennan *et al.*, 2003). In addition, the students, especially undergraduate students, voice the need for more academic support, and suggest strengthening the advising skills of their instructors. Gaps in the teaching styles and methods of some instructors were also indicated. There seems to be a need to enhance the overall organization of courses, and the quality of student performance and progression assessment methodologies, as well.

As reflected upon in the results, the high level of perceived respect and support can be attributed to the opportunities that the students are offered to express their opinions. It is believed that engaging students contributes to enhancing their learning experience, through nurturing the connection between the instructors and their students ( Cook-Sather, 2014; Cook-Sather and Felten, 2017). Along the same lines, Keskitalo and Ruokamo (2016) propose that the sense of belonging, among the students and the faculty members, fosters a cycle of deepening engagement and in turn, meaningful development. When students see themselves as active partners in their own educational experience, they become more focused and invested in their learning efforts ( Cook-Sather, 2016). Listening to the voice of learners also enables them to feel safe in their environment which feeds into their overall sense of contentment.

It is worth acknowledging that this evidence-based system of engaging students at MBRU in identifying the development needs of their instructors is still at a developmental phase. It would be worthwhile, from a practical perspective, to run a follow-up study that maps the same information onto the perception of the instructors themselves in relation to their own professional development needs. Longitudinal studies, around the same variables, will also be of added value, to assess the long-term impact of such CQI models on the professional development trajectory and the performance of faculty members. All this can provide more evidence, concerning setting-up a similar CQI system, for other institutions to base their decisions upon. Moreover, although this study offers beneficial insights for other tertiary education settings, the generalizability of its results, when it comes to the pinpointed faculty development needs, are limited to institutions that are contextually and characteristically similar to MBRU. Hence, it will be useful for future studies to investigate faculty development needs, from the perspective of the ultimate receivers: the students, across several institutions.

## Conclusion

The study concluded that there is added value in capturing and effectively assembling the learners’ perception of faculty performance. It raises the reliability of the implemented evaluation framework, and has the potential of improving the rigor of faculty development initiatives. The study also recommended deploying a pragmatic technique to consolidating insights from undergraduate and postgraduate students in order to leverage the perceptive value of the feedback, which is expected to better inform faculty development initiatives.

## Take Home Messages

• There is added value in capturing and effectively assembling the learners’ perception of faculty performance.
• Capturing the learners’ perception of faculty performance enables tackling all four levels of the Kirkpatrick model, which is expected to raise the reliability of the adapted evaluation framework.
• Such an evidence-driven Continuous Quality Improvement system has the potential of improving the rigor of faculty development initiatives.
• The undergraduate and postgraduate students offer differing yet complimentary perspectives.
• The high level of perceived respect and support can be attributed to the opportunities that the students are offered to express their opinions.

## Notes On Contributors

**Farah Otaki:** Senior Specialist- Strategy and Institutional Excellence, Mohammed Bin Rashid University of Medicine and Health Sciences, Dubai, United Arab Emirates. ORCID ID: https://orcid.org/0000-0002-8944-4948

**Amar Hassan Khamis:** Professor- Biostatistics, Hamdan Bin Mohammed College of Dental Medicine, Mohammed Bin Rashid University of Medicine and Health Sciences, Dubai, United Arab Emirates

**Reem AlGurg:** (1) Director- Strategy and Institutional Excellence, and (2) Assistant Professor, Health Policy, College of Medicine, Mohammed Bin Rashid University of Medicine and Health Sciences, Dubai, United Arab Emirates

**Mohamed Nasaif:** Training and Development Consultant, Ministry of Health and Prevention, United Arab Emirates

**Dave Davis:** Senior Director, Centre for Outcomes and Research in Education, Mohammed Bin Rashid University of Medicine and Health Sciences, Dubai, United Arab Emirates

**Nabil Zary:** (1) Director, Institute for Excellence in Health Professions Education, and (2) Professor, Medical Education, Mohammed Bin Rashid University of Medicine and Health Sciences, Dubai, United Arab Emirates

## References

[ref1] Baruch Y. (1999) Response rate in academic studies- A comparative analysis. Human Relations. 52. https://doi.org/10.1177/001872679905200401

[ref2] Baruch Y. and Holtom B. C. (2008) Survey response rate levels and trends in organizational research. Human Relations.pp.1139–1160. 10.1177/0018726708094863

[ref3] Beckman T. J. Ghosh A. K. Cook D. A. Erwin P. J. (2004) How Reliable Are Assessments of Clinical Teaching? A Review of the Published Instruments. Journal of General Internal Medicine. 19(9), pp.971–977. https://doi.org/10.1111/j.1525-1497.2004.40066.x 15333063 10.1111/j.1525-1497.2004.40066.x PMC1492515

[ref4] Bennett D. Dornan T. Bergin C. and Horgan M. (2014) Postgraduate training in Ireland: expectations and experience. Ir J Med Sci. 183(4), pp.611–620. 10.1007/s11845-013-1060-5 24390312

[ref5] Berbano E. P. Browning R. Pangaro L. and Jackson J. L. (2006) The impact of the Stanford Faculty Development Program on ambulatory teaching behavior. J Gen Intern Med. 21(5), pp.430–434. 10.1111/j.1525-1497.2006.00422.x 16704383 PMC1484783

[ref6] Bewley W. L. and O’Neil H. F. (2013) Evaluation of medical simulations. Mil Med. 178(10 Suppl), pp.64–75. 10.7205/MILMED-D-13-00255 24084307

[ref7] Bovill C. Cook-Sather A. and Felten P. (2011) Students as co‐creators of teaching approaches, course design, and curricula: implications for academic developers. International Journal for Academic Development. 16(2), pp.133–145. 10.1080/1360144X.2011.568690

[ref8] Brennan J. Brighton R. Moon N. Richardson J. (2003) Collecting and using student feedback on quality and standards of learning and teaching in Higher Education. A report to HEFCE by the Centre for Higher Education Research and Information (Open University), NOP Research Group and SQW Ltd. https://dera.ioe.ac.uk/4988/1/rd08_03.pdf( Accessed: 12/08/2020).

[ref9] Burdick W. P. Diserens D. Friedman S. R. Morahan P. S. (2010) Measuring the effects of an international health professions faculty development fellowship: the FAIMER Institute. Med Teach. 32(5), pp.414–421. 10.3109/01421590903394587 20423261

[ref10] Clay M. A. Sikon A. L. Lypson M. L. Gomez A. (2013) Teaching while learning while practicing: reframing faculty development for the patient-centered medical home.. Academic Medicine. 88(9), pp.1215–1219. https://doi.org/10.1097/ACM.0b013e31829ecf89 23887006 10.1097/ACM.0b013e31829ecf89

[ref11] Cook-Sather A. (2014) Multiplying perspectives and improving practice: What can happen when undergraduate students collaborate with college faculty to explore teaching and learning. Instructional Science. 42(1), pp.31–46. 10.1007/s11251-013-9292-3

[ref12] Cook-Sather A . (2016) Creating brave spaces within and through student-faculty pedagogical partnerships. Teaching and Learning Together in Higher Education. 1(18). https://repository.brynmawr.edu/cgi/viewcontent.cgi?article=1143&context=tlthe( Accessed: 12/08/2020).

[ref13] Cook-Sather A. and Felten P. (2017) Where student engagement meets faculty development: How student-faculty pedagogical partnership fosters a sense of belonging. Student Engagement in Higher Education Journal. 1(2), https://pdfs.semanticscholar.org/7e15/5009dbf065d2774dbef797d58d0b083ca09b.pdf( Accessed: 12/08/2020).

[ref14] Ginns P. Prosser M. and Barrie S. (2007) Students’ perceptions of teaching quality in higher education: the perspective of currently enrolled students. Studies in Higher Education. 32(5), pp.603–615. 10.1080/03075070701573773

[ref15] Keskitalo T. and Ruokamo H. (2016) Students’ expectations and experiences of meaningful simulation-based medical education. International Seminar. 12(2), https://journals.hioa.no/index.php/seminar/article/view/2331/2148( Accessed: 12/08/2020).

[ref16] Kirkpatrick D. L. and Kirkpatrick J. D. (2006) Evaluating training programs: the four levels. Williston: Berrett-Koehler. https://doi.org/10.1097/NND.0000000000000077

[ref17] Lindsay R. Breen R. and Jenkins A. (2010) Academic Research and Teaching Quality: The views of undergraduate and postgraduate students. Studies in Higher Education.pp.309–327. https://doi.org/10.1080/03075070220000699

[ref18] Little S. (2004) -isms: Understanding Art. Universe Pub. https://agreatread.co.uk/isms-understanding-art-new-edition-9781912217212/( Accessed: 12/08/2020).

[ref19] Morton S. M. Bandara D. K. Robinson E. M. and Carr P. E. A. (2012) In the 21st Century, what is an acceptable response rate? Aust N Z J Public Health. 36(2), pp.106–108. 10.1111/j.1753-6405.2012.00854.x 22487341

[ref20] Motola I. Devine L. A. Chung H. S. Sullivan J. E. (2013) Simulation in healthcare education: a best evidence practical guide. AMEE Guide No. 82. Med Teach. 35(10), pp.e1511–e1530. 10.3109/0142159X.2013.818632 23941678

[ref21] Mundy D. (2002) A question of response rate. Science Editor. 25, pp.25–26, http://citeseerx.ist.psu.edu/viewdoc/download?doi=10.1.1.673.3284&rep=rep1&type=pdf( Accessed: 12/08/2020).

[ref22] Nulty D. D. (2008) The adequacy of response rates to online and paper surveys: what can be done? Assessment & Evaluation in Higher Education. 33, pp.301–314. 10.1080/02602930701293231

[ref23] O’Sullivan P. S. and Irby D. M. (2011) Reframing research on faculty development. Acad Med. 86(4), pp.421–428. 10.1097/ACM.0b013e31820dc058 21346505

[ref24] Ramsden P. (2006) A performance indicator of teaching quality in higher education: The Course Experience Questionnaire. Studies in Higher Education.pp.129–150. 10.1080/03075079112331382944

[ref25] Samarakoon L. Fernando T. Rodrigo C. and Rajapakse S. (2013) Learning styles and approaches to learning among medical undergraduates and postgraduates. BMC Med Educ. 13, p.42. 10.1186/1472-6920-13-42 23521845 PMC3620557

[ref26] Steinert Y. (2017) Faculty Development: From Program Design and Implementation to Scholarship. GMS J Med Educ. 34(4),p. Doc49. 10.3205/zma001126 PMC5654113 29085893

[ref27] Stewart G. (2003) Literary impressionism and modernist aesthetics. Cambridge University Press. https://doi.org/10.1353/mod.2003.0089

[ref28] Ten Cate O. Mann K. McCrorie P. Ponzer S. (2014) Faculty development through international exchange: the IMEX initiative. Med Teach. 36(7), pp.591–595. 10.3109/0142159X.2014.899685 24787528

[ref29] Wright D. B. Mullen A. and Gardner A. (2019) Does Student-Led Faculty Development Have A Place in Health Professions Education? MedEdPublish, 8. 10.15694/mep.2019.000034.1 PMC10712635 38089393

[ref30] Young M. (2015) Realism in the Age of Impressionism: Painting and the Politics of Time. Yale University Press. 10.1086/691477

[ref31] Zamora L. P. and Faris W. B. (1995) Magical realism: Theory, history, community. Duke University Press. https://doi.org/10.2307/j.ctv11cw5w1

