# Supplementary material for: Exploring the value of the learners’ perception of teaching effectiveness in informing faculty development needs: A mixed-methods study
Source: MedEdPublish (2016). 2020 Sep 30;9:213. [Version 1] doi: 10.15694/mep.2020.000213.1 (PMC10699389; doi:10.15694/mep.2020.000213.1)
Supplement: Peer review history

# Reviewer report: Reviewer response for version 1

**DOI:** 10.21956/mep.20157.r31142

**Published:** 2020-10-12

**Authors:** Mohamed Al-Eraky

**Affiliations:** 1 University of Dammam

This review has been migrated. The reviewer awarded 4 stars out of 5 This interesting study aimed to solicit learners’ perception of faculty performance as a tool to measure the effectiveness of faculty development programs. I would concur with the authors that it’s a great approach. Yet, I would like to advocate that ensuring effectiveness starts from planning, before induction. We used to plan our programs based on the interests (wishes, not needs) of staff, but we need to acknowledge three other inputs to the plan, as indicated in the Compass Model (Figure 1) in here: https://drive.google.com/file/d/0ByugEmLvxFlUaGQydTBBX0h2czQ/view?usp=sharing There is a tricky point in needs assessment. We should fulfill the actual needs, not wishes. That’s why surveys are not enough, we need valid assessment of the performance of potential participants to be considered in planning, implementation and eventually of evaluation of the effectiveness of any faculty development program. Good luck.

Reviewer Expertise:

NA

No decision status is available

# Reviewer report: Reviewer response for version 1

**DOI:** 10.21956/mep.20157.r31143

**Published:** 2020-10-05

**Authors:** P Ravi Shankar

**Affiliations:** 1 American International Medical University

This review has been migrated. The reviewer awarded 4 stars out of 5 This is an interesting article exploring student feedback in faculty development programs at a medical school in the United Arab Emirates. The authors have used data from both the quantitative and qualitative feedback obtained from both postgraduate dental and undergraduate medical students. Among the areas requiring improvement were teaching skills of some faculty members, better communication between faculty and students, strengthening academic advising, and greater student involvement in their learning. The process of collecting and analysing the feedback is well described. The authors could provide a more concrete description of how they plan to use the survey findings to strengthen faculty development at their institution. Were they able to obtain specific responses from the students which may be useful regarding designing specific faculty development initiatives? This is a well written manuscript which will be of general interest.

Reviewer Expertise:

NA

No decision status is available
